# A Case of Gangrena Oris

**Published:** 1883-03

**Authors:** D. A. Hengst

**Affiliations:** Pittsburgh, Pa.


### ARTICLE IX.

### *A Case of Gangrena Oris.*

BY D. A. HENGST, M. D., OF PITTSBURGH, PA.

On July 28, 1883, I was called to see Mary N. White, two years of age, with the following history: She had just recovered from a moderately severe attack of measles, and now had a swollen, pale face, œdema about both eyes, but the swelling was hard and painful over both parotid and sub-maxillary glands, and of a shining, glistening appearance; the mucous membrane of the mouth was also swollen and inflamed. Her surroundings were those of poverty and filth, and she had the appearance of an ill-nourished and badly-fed child. Her general condition was bad—pulse 120, temperature about 102°, tongue coated, had some diarrhœa, and was very restless and irritable. The next day the swelling had become more marked. She had indurated patches on the scalp over left parietal bone, at the occiput; over the glands of the face, on lower lip, and on the upper border of the sternum. About two days after this, eschars formed over all these indurated spots as well as on the mucous membrane of the mouth at a point opposite the last molar tooth on each side. In about one week after my first visit these eschars had sloughed away, leaving deep ulcers, which continued to spread in width and depth until in some of them all the soft tissues were destroyed to the bone. She now presented a most pitiable appearance. On the scalp were two large ulcers about the size of a silver dollar, exposing the bone over its entire base; in a short time the narrow bridge of tissue between them was eaten away, thus exposing almost the entire left parietal bone; back of this, over the occiput, was another extending to the bone and about one inch in diameter. Over both parotid glands were deep ulcers from an inch to an inch and a half in diameter, and of a circular shape. A large section of the lower lip had sloughed away, making it appear like a harelip, and causing considerable difficulty in deglutition. The ulcer on the upper border of the sternum, about three-fourths of an inch in diameter, was the most destructive one, for at the time of her death it had ulcerated its way into the pleural cavity. All of these ulcers had tough and sharply-defined edges, with no inflammatory areola. Those on the inside of the mouth were not as deep or destructive as the external ones. Her general condition now was very bad. Pulse very rapid, high fever every day, with diarrhœa and cough. She continued in this condition until the 23d day of August, when a severe convulsion caused her death.

As regards treatment, when the case was first seen it looked like one of erysipelas of the face, and I prescribed accordingly; but as soon as the eschars formed I began to suspect the terrible nature of the disease I had to deal with.

Then applied strong nitric acid as a cauterant and alterative; repeated this at various times, and afterwards used a paste of carbolized water; but these applications would not check the spreading tendency of the disease, nor change the character of the ulcers.

Internally she was given morphine when needed to calm irritability. Tr. Ferri Chlor., Quinia and stimulants, also nutritious food, such as beef tea, milk, etc This has been to me a very interesting as well as an extremely rare disease, being the first case of the kind in a practice of thirteen years. The slow progress of the disease, and its not affecting the mouth as much as the tissues on the outside, might be brought forward as an argument against gangrene of the mouth. But the course of the disease is not always so rapid. In consulting the authorities upon this subject, I find it described as being extremely rare in private practice, but more frequent in hospitals for children; that unfavorable hygienic conditions constitute a predisposing cause, and that it almost always follows upon some acute or chronic disease, especially measles. In this case the hygienic surroundings were extremely bad, and it followed an attack of measles, M. Baron says he has never known it to occur as an idiopathic disease, and that it is questioned whether or not large and continued doses of mercury have ever produced it. —*Med. and Surg. Reporter.*

---

